# Insights into the Social Structure of the PPNB Site of Kfar HaHoresh, Israel, Based on Dental Remains

**DOI:** 10.1371/journal.pone.0134528

**Published:** 2015-09-16

**Authors:** Kurt W. Alt, Marion Benz, Werner Vach, Tal L. Simmons, A. Nigel Goring-Morris

**Affiliations:** 1 Center of Natural and Cultural History of Teeth, Danube Private University, Krems-Stein, Austria; 2 Integrative Prehistory and Archaeological Science IPAS, Basel University, Basel, Switzerland; 3 State Office for Heritage Management and Archaeology Saxony-Anhalt and Heritage Museum, Halle, Germany; 4 Department of Near Eastern Archaeology, Albert-Ludwigs University, Freiburg, Germany; 5 Center of Medical Biometry and Medical Informatics, University Medical Center, Albert-Ludwigs University, Freiburg, Germany; 6 Institute for Advanced Studies, The Hebrew University of Jerusalem, Jerusalem, Israel; 7 Institute of Archaeology, The Hebrew University of Jerusalem, Jerusalem, Israel; Museo Nazionale Preistorico Etnografico 'L. Pigorini', ITALY

## Abstract

One of the central questions of the transition from mobile hunter-gatherers to sedentary farming communities concerns the establishment of new social structures and group identities. Along with other important factors, such as territory, ideology or economy, biological relationships might have played a decisive role in defining social groups. We therefore systematically analyzed teeth and jaw remains from nine sites in the Near East dating from the Natufian to the Late PPNB as primary proxy data for the reconstruction of familial relationships. This paper presents the results of morphological analyses on the teeth of the individuals from Kfar HaHoresh, one of the investigated Pre-Pottery Neolithic B sites. Kfar HaHoresh is located in the Nazareth hills of Galilee (32°42'20'' N 35°16'28'' E), Israel. Different statistical methods were applied to our data of epigenetic traits with the aim of determining biological relationships within the community, whereby the data of the eight other sites were used as cross-references. Our comparison of the traits of all individuals from Kfar HaHoresh indicates a rather heterogeneous community, but clearly shows one cluster belonging to a quite homogenous group, suggesting close biological relations between females and sub-adults. Interestingly, none of the male individuals belongs to this cluster, although their number outweighs that of the female individuals. This might suggest matrilocal residence patterns. However, due to the incomplete preservation of the teeth along with several other uncertainties, our conclusion must be seen as preliminary. A cross-examination of the results on skeletons excavated after our investigation should also be taken into consideration.

## Introduction

In the Near East, between the 13^th^ and 10^th^ millennium BC, one of the most fundamental processes started that would change human life forever [1–4]. Hunter-gatherers settled down permanently and established large agglomerations of hitherto unimaginable scale (for a summary and critical discussion see [5–6]). This process had an impact on the social organization and identities comparable only with the later processes of urbanization, industrialization and digitalization. Several authors have attempted to reconstruct the social changes that accompanied sedentarization and the beginnings of cultivation and herding. They have argued for the emergence of newly defined socio-political entities like sodalities, age groups [7–8], and extended families [9–10], cf. [11]. The specific term of *extended family* was replaced by Nissen et al. [12] by the less restrictive description of “new corporate large family structure”. In contrast, Flannery [11] argued for a shift from communal extended families to nuclear families based on the shift from round huts of the Natufian and PPNA to rectangular buildings of the PPNB. He later added a third stage with a transition to extended families during the Pottery Neolithic [13]. Based on the emergence of multi-roomed architecture, communal buildings, an increase in symbolic devices and a differentiation of burial customs in the Levant especially during the Middle and Late Pre-Pottery Neolithic B, between the 9^th^ and 8^th^ millennium cal BCE, increasing social differentiation of early Holocene communities has been suggested (*e*.*g*. [14]; see also the papers in [15]; and [16–19]). Due to the wide range of possible interpretations, especially the multi-functionality of architecture, these studies remain hypotheses to be tested. Belfer-Cohen and Goring-Morris [20] have pointed to local traditions of body decoration and burial traditions as early as during the Natufian. It therefore remains an open question whether–and if so, how–the social structure of communities changed at the transition to sedentary life before farming [21]. In light of these questions, the major focus of the present study is aimed at analyzing the human remains from Kfar HaHoresh. This is connected to the site’s supposed special character as a periodically used cult-and-mortuary feasting center [22–24], cf. [25–26]. If this interpretation holds true, the question is who came here to feast and bury their dead? Was it a familial community? A special group (ritual elite; secret sodalities?)?; or, could the site be accessed by everybody to bury their dead?

Only the human remains can provide possible answers, but despite their high value as proxies for archaeological interpretations, they have until recently been used only rarely for gaining systematic information on social or familial structures in the prehistoric Near East [27–31] (for systematic approaches in European Archaeology see [32–34]). Insufficient preservation of ancient DNA [35–38] as well as financial, structural and logistic difficulties of post-excavation meta-studies are the most important reasons.

The SIGN Project was therefore an invaluable chance to systematically integrate skeletal remains as primary proxy data for the reconstruction of social identities. Including the Early-to-Late PPNB site of Kfar HaHoresh, the corpus consists of nine sites dating from the Natufian to the Late PPNB with which our data can be cross-referenced. However, the poor and fragmentary preservation (almost no maxillary teeth were available for analyses) means a considerable bias in our data for Kfar HaHoresh and requires utmost caution in the interpretation. Post-depositional taphonomic processes have resulted in better preservation of the more robust mandibles as opposed to the crania. In addition, secondary burials were more common at Kfar HaHoresh than primary interments, the latter often having had the skull removed.

Additionally, in 2007, when the data were collected, only a part of the now available skeletons had been excavated [39] (ANG-M pers. obs.). Detailed data on osteological characteristics, mobility, diet, stress markers and pathologies should complete the picture in the future and may lead to reconsideration of some of our interpretations.

### Archaeological and Anthropological Background

**Fig 1:**
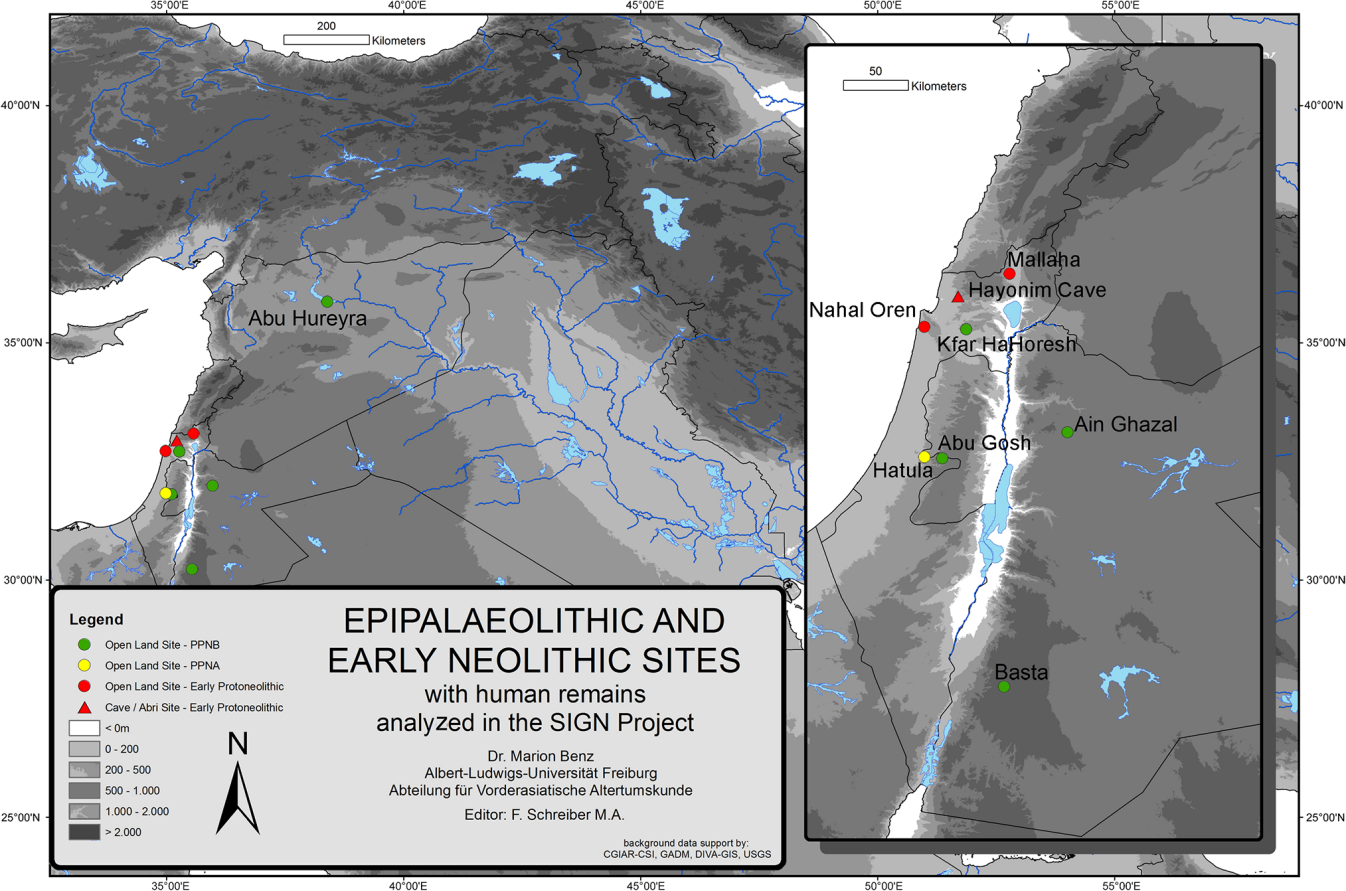
Sites with human remains analyzed in the SIGN-Project [31].

The epigenetic traits of 21 individuals from Kfar HaHoresh have been analyzed in the framework of the SIGN Project [7, 31] (Fig 1). Including Kfar HaHoresh, data from nine sites from the Natufian to the Late PPNB were collected: Hayonim Cave, Nahal Oren and Mallaha for the Natufian, Hatula as the only PPNA site, Abu Gosh, Ain Ghazal, Abu Hureyra, Basta and Kfar HaHoresh for the PPNB. Precise location and radiocarbon data for all sites are given in the open access data base PPND [40]. Although the number of analyzed individuals from Kfar HaHoresh is statistically at the lower limits for a meaningful intra-population analysis, it is an important site due to its postulated special character.

**Fig 2:**
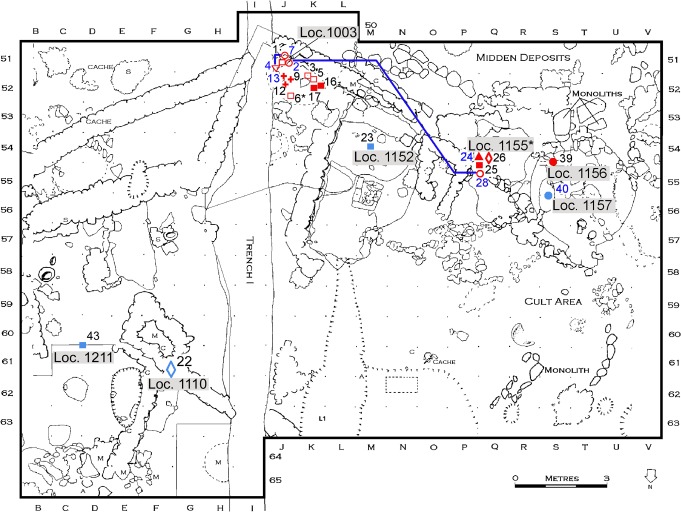
Plan of Kfar HaHoresh (modified after [22]). Loci with human remains analyzed in this study: blue numbers indicate related individuals in Group A/B, blue line indicates individuals related according to the distance analysis. *Loc. 1155 includes Loci 1352, 1353 and 1373. Blue colored = middle occupational period red colored = late occupational period filled / unfilled square = male / male? Adult; filled / unfilled circle female/female? Adult; unfilled diamond = adult indet; filled / unfilled triangle pointing upwards = male/male? juvenile, filled / unfilled triangle pointing downwards = female/female? Junvenile; cross = infans.

The excavations at Kfar HaHoresh have been ongoing since 1991 [22, 24, 39, 41–43]. Techno-typological characteristics of the chipped stone tool assemblages and radiocarbon dates suggest an occupation from the Early to the Late PPNB (ca. 8750–7500 cal BCE) [39], the human remains we analyzed all date from the middle to late periods of occupation (Fig 2 and Table 1). Due to its small size of less than 1 ha, its unusual secluded location on the northern incline of a narrow valley on the western flanks of the Nazareth hills, but with a nearby panoramic view, the special architectural features and ritual remains, a lack of unequivocal domestic structures and the many burials, Kfar HaHoresh has been interpreted as a cult and funeral center [22, 24, 44–45]. The particular demographic characteristics seem to support this interpretation. “The Kfar HaHoresh mortality curve is characterized by a high mortality rate between the ages of 20 to 29 years. This unusual demographic profile accords well with other lines of evidence indicating that Kfar HaHoresh may represent a regional ritual/cultic mortuary centre” [46]. However, there are several similarities with other Middle and Late PPNB sites of the Levant, with burials beneath plastered floors/surfaces, a high variability in primary and secondary burial customs and, above all, the plastered skulls, e.g. [7, 19, 26, 47–51]. Despite recent discussion concerning its interpretation as a special cult center [25], the extraordinary ritual activities at Kfar HaHoresh are undeniable.

**Table 1:** Archaeological and relevant anthropological data of the analyzed individuals from Kfar HaHoresh.

ID N°	#—fieldnumber	Location number	Period	Sex	Age in Years	Age groups	Evaluableteeth—maxilla	Evaluableteeth-mandible	Evaluableteeth in total^1^
1	**#**25	1003, J51d	L	Male? [Male]	25–30 [40–44]	Adult [Mature]		13	13
**2**	**#26**	**1003, J51d**	**L**	**Female? [?]**	**20–25**	**Adult**		**3**	**3**
3	**#**226	1003, K52a	L	Male? [?]	25–30 [30–34]	Adult		3	3
**4**	**#293**	**1003, J51c**	**L**	**Female? [?]**	**15–20**	**Juvenile**		**10**	**10**
5	**#**642	1003, K52	L	Male? [Male]	25–30 [40–44]	Adult [Mature]		9	9
6	**#**689	1003,	L	Male? [Male]	25–30 [40–44]	Adult [Mature]		4	4
**7**	**#571/586 [575]**	**1003, J51a**	**L**	**Female? [Male]**	**15–25 [20**–**24]**	**Adult**		**12**	**12**
9	**#**279	1003, J52a	L	?	5–9	Infans II^2^		2	2
12	**#**591	1003, J52a	L	?	7–9	Infans II		2	2
**13**	**#640**	**1003, J52**	**L**	**?**	**4–6 [5**–**9**]	**Infans II**	**1**	**7**	**8**
16	**#**133	1003, K52b	L	Male	20–24	Adult		9	9
17	**#**135	1003, K52b	L	Male	20–24	Adult		3	3
22	1 **#**4	1110/1114F61d	M	?	35–40 [40–44]	Adult [Mature]		2	2
23	1	1152/1250M54a	M	Male	20–24	Adult	1	3	4
**24**	**H1#82 #128**	**1155/13521353/1373**	**L**	**Male**	**15**	**Juvenile**		**11**	**11**
25	H2**#**325	1155/13521353/1373	L	Male	35–45 [20–24]	Adult		6	6
26	H3 **#**472	1155/13521353/1373 Q58a/b	L	?	25–30 [3–5]	Adult [Infans I]		2	2
**28**	**H5#808**	**1155/13521352/1373**	**L**	**Female? [Male]**	**25–35 [20–24]**	**Adult**		**15**	**15**
39	2 **#**29	1156	L	Female	20–24	Adult		10	10
**40**	**1 #28**	**1157 S56/R56**	**M**	**Female**	**25–30**	**Adult**		**6**	**6**
43	1 **#**18-452-35	1211/1109F160a	M	Male	30–34	Adult	3		3

# = after [46] and Kranzbühler/Simmons pers. obs.; age and sex data = after Simmons; age and sex determinations by [46] are given in square brackets where differences occurred. Bold type indicates individuals belonging to Group A or B.

^1^ teeth are documented by photographs (S1–S3 Figs).

^2^ age determination by [46] in accordance with the photo.

The four most ancient human remains that were analyzed in this study come from four different locations: Loci 1153, 1157, 1211, and 1110, in the production area (Squares C-G 60–62) and cult area (Squares L-M/53 and S-T/55-56) (Fig 2). They were dated to the middle phase of the occupation.

More than half of the analyzed individuals (n = 12) come from Loc. 1003, a “kidney-shaped ashy burial pit” of about one and a half meter in diameter, beneath the lime plastered surface of Loc. 1001. Two headless primary burials were found at the bottom of the pit. A number of mostly disarticulated human and animal bones, including 13 mandibles, were found above these individuals [44]. Stratigraphically speaking, Loc. 1003 belongs to the late period of occupation.

Five other individuals belong to the late occupation phase. Four of them were discovered in the area of one of the most curious features of the site—the “animal depiction”; Loci 1155, 1352, 1353, and 1373 (Squares Q-R/54-55) [52]. Beneath a plastered surface (Loc. 1027), numerous human bones were intentionally arranged in a shallow ash pit, perhaps depicting an animal. The fifth individual (ind. 39) of the late period was discovered in Loc. 1156 (Square Q54), sealed by a patch of plaster immediately above the surface of Loc. 1027, and thus directly overlying the Loc. 1155 complex [44].

## Material

Up until 2007, when the data for this analysis were collected, the minimum number of individuals discovered varied between 30 and 54 individuals [44, 46]. Today, it amounts to about 80 individuals [39, 42]. The site was occupied from the Early PPNB through the beginning of the Late PPNB based on techno-typological aspects of the lithic assemblages and supported by a series of C14 dates, including the Loc. 1003 and Loc. 1155 grave pits dating to ca. 7,500/7,700 cal BCE. Due to the rather poor preservation of teeth and jaws, only 21 individuals (30%) were evaluable for a morphological analysis of biological relationships. Full details concerning specimen numbers and proveniences at Kfar HaHoresh are provided in Table 1 (columns 2, 3, and 4). The human remains from the excavations at Kfar HaHoresh, including all of those examined for the present study, are presently stored in the Laboratory of Anatomy and Anthropology of the Sackler School of Medicine, Tel Aviv University, Ramat Aviv, Israel. The Israel Antiquities Authority (IAA) issued the excavation licenses at Kfar HaHoresh–the site is listed in the IAA archives as Site 6023/0 (IG refs. 225600-734400/225700-734500)–in the name of A.N. Goring-Morris only on an annual basis for each of the field seasons from 1991. The licenses for the various field seasons conducted at Kfar HaHoresh include: License #G-60/2012, License #G-43/2011, License #G-29/2010, License #G-39/2009, License #G-52/2008, License #G-73/2007, License #G-46/2004, License #G-82/2002, License #G-84/2001, License #G-78/2000, License #G-123/1999, License #G-104/1998, License # G-108/1997, License #G101-1996, License A-1992/1893, License #1991/1801. The licenses are filed in the archives of the Israel Antiquities Authority in Jerusalem. No further permits beyond those described above were required for the present study.

The data set comprises 18 adolescent-to-adult individuals and three children. Two of the adolescent and adult individuals were determined as certainly female, four as probably female, six as male and four as probably male. The sex distribution is thus slightly biased towards male individuals (6:10, Fig 3). For two of the adult individuals and for the three children, a sex determination was not possible.

**Fig 3:**
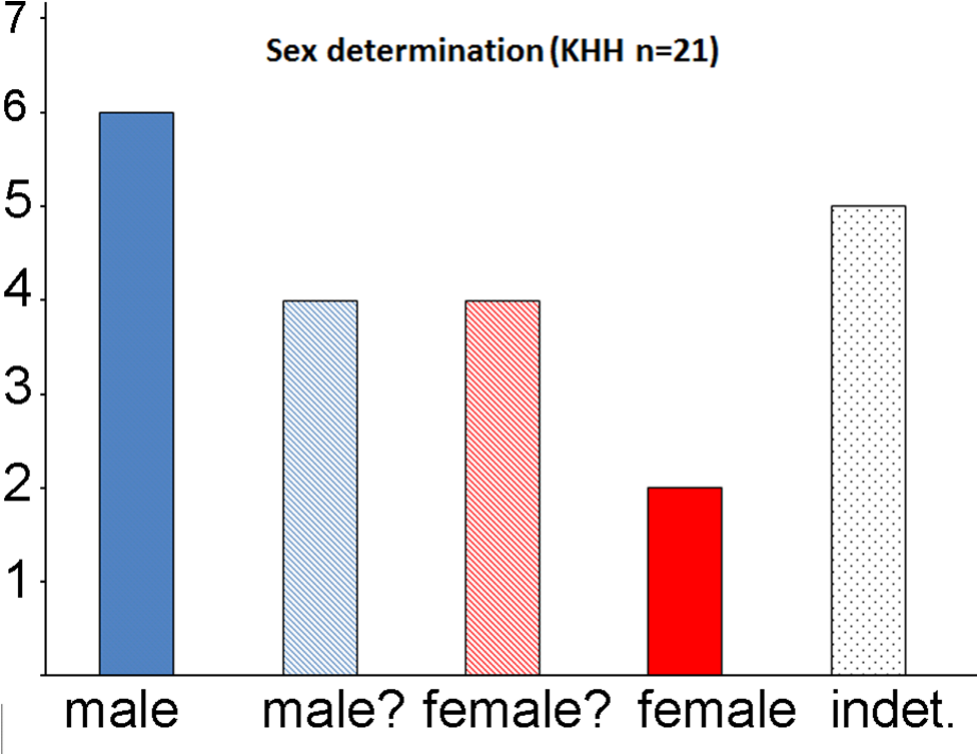
Sex distributions of the investigated individuals. n = absolute numbers (based on data from Eshed et al. [46]; modified after Simmons S1 Table).

A former determination of the sex of the individuals was based on the innominate bone, the long bones and skulls mainly. However the pelvis was so badly preserved that only one individual of each sex could be determined on the basis of this bone [46]. Skulls were often separated from the postcranial remains such that a secure attribution to a certain individual was difficult. Tal L. Simmons therefore based her analyses on the well preserved, ubiquitous and thus comparable jaws, which were available for most of the individuals. Thus, the analyses of both observers do not compete but complement each other. Due to intra-population variability of the jaws, it was possible to determine the sex and age of some individuals more precisely.

We therefore consider the sex determination and the age estimation according to the jaws and teeth as an addition and specification to the former investigator’s results (S1 Table). Except for two individuals (ind. 7 and ind. 28) and two obvious reversed individuals (ind. 9 and ind. 26; S1–S3 Figs), most of the sex determinations of both observers match quite well (Fig 3 and S1 Table) [46].

Simmons’ determinations of age and sex were based on the following characteristics as outlined in Buikstra and Ubelaker [53]. Mandibles were judged to be male if they exhibited overall robust morphology, strongly marked insertions for the medial pterygoid and masseter muscles, and double-pointed (square) mandibular symphyses; mandibles were judged to be female if they exhibited overall gracile characteristics and a single-pointed mandibular eminence. Dental age estimation for juveniles was based on crown and root development as well as dental eruption sequences and, for adults, on dental wear patterns. For individuals not analyzed or undetermined by Simmons we applied the results given by Eshed et al. [46].

The teeth of the upper and lower jaw were evaluable for only two individuals. The teeth of the mandible were evaluable for 18 individuals and for one individual only the teeth of the maxilla were evaluable. The comparative dental index (a measure of the number of teeth evaluable) of 22% on average reflects the incomplete preservation of teeth. The basic archaeological and anthropological data of the studied individuals are summarized in Table 1. As reference populations we used the epigenetic data of the other eight investigated sites of the SIGN-Project (joint dataset).

## Methods

Generally speaking, morphological and genetic methods can be applied for the identification of biological relationships in archaeological burial contexts. Molecular genetic methods can only be applied to ancient DNA (aDNA), if the preservation of the skeletal remains is very good. This is only rarely the case in prehistoric and historic populations [32, 37]. Long before molecular a-DNA analyses became available, many investigators started to study anatomical variants on skeletons (*epigenetic traits*), searching for biological relationships in past populations [54–55]. However, odontological traits fulfill the requirements for kinship studies to a much higher degree than skeletal markers [56]. Dental traits are easily validated in living populations, and information on the heredity of many traits is available [57–58]. In order to obtain suitable results for the analysis of relationships, anatomical characteristics have to satisfy certain requirements, such as a low frequency of occurrence within the population, a manifestation that is easily recognized and detectable with simple techniques, low variability in respect to age and sex, and independent occurrence from other characteristics used in studies [59–60]. However, most important is the high heritability of the epigenetic traits, e.g. [61]. Most discrete traits are polygenically controlled, and the exact mechanisms of their heredity are known only to a certain degree [56, 58], but as Scott [62] summarized:”Twin and family studies indicate dental morphology has a strong heritable component“. Practical experience has shown that dental characteristics are best at meeting these requirements [63–64].

The geographic variation in tooth crown and root characteristics is therefore well known as a powerful tool in dental anthropology for both inter- and intra-population analyses. Inter-population studies are most used to reconstruct population history as well as continental and regional differentiation of past human groups (last by [65–66]). Infra-population respectively intra-cemetery evaluates as presented in this study has been tested in communities with diverse burial customs using different approaches [56, 67–69]. It is assumed that groups of individuals of both sexes and all ages buried together in burial communities such as collective graves, grave fields or other burial units generally represent members of local populations. These social communities comprise subgroups of individuals displaying a relatively high biological or rather genetically similarity due to common descent. The idea that biologically related individuals have a number of phenotypical traits (syn. epigenetic traits, anatomical variants) in common that are typical for their family is the key premise for reconstructing such subgroups from skeletal remains [70]. These characteristics have to be separated from a multitude of anthropological features present on the material. The results are family groups consisting of genetically related individuals. In such studies the terms “family” and “relationships” are therefore understood in the biological sense as opposed to the broader social concept encompassing all members of a house and living community (incl. servants, relatives by marriage, etc.).

More than 1000 traits suitable for use in kinship analysis have been catalogued [64]. The list consists of four types of traits: variants of the tooth crown and roots, dental anomalies of the shape, number, size, structure and position of teeth, selected non-metric traits of cranium, maxilla and mandible, and congenital malformations and syndromes involving jaws and teeth. As most of the traits are investigated on all teeth, the number of traits is very high. The approaches for the detection of biological relationships and families and the statistical procedures to validate the results are described in detail in numerous publications [71–74].

For the analyses of the skeletal remains from Kfar HaHoresh, we used three search strategies [64]. Strategy 1: Search for increased traits frequencies in the sample population; Strategy 2: Search for pairwise similarities and Strategy 3: Search for conspicuous groups of individuals within the data matrix. Strategy 1 is based on the expectation that some traits are of such high relative frequency within a family that the whole sample population differs in frequency of the trait to the overall population. For Strategy 2, it is expected that related individuals show up by common traits and hence show closer similarities than non-related individuals. In Strategy 3 biological relationships should be demonstrated by the detection of groups of several individuals who share at least two or more common traits, whereas outside these groups the frequency of these traits is low.

Unfortunately, in the study at hand, the starting point for such analyses is rather poor. Only 29 traits could be identified that were expressed in at least two individuals from Kfar HaHoresh. Table 2 lists these traits and only these 29 traits could contribute to the further analyses.

**Table 2:** List of traits evaluated for the analyses of biological relationship on teeth from Kfar HaHoresh.

	Traits no.	Description of the traits^1^	Evaluable (n = 21) frequency in %	Relative frequency in %
1	7	Lingual Surface flat—31 41	3 = 14,3	3 = 100
2	9	Lingual Surface flat—32 42	3 = 14,3	3 = 100
3	11	Lingual Surface flat—33 43	8 = 38,1	8 = 100
4	15	Lingual Surface flat—31 41 32 42	5 = 23,8	5 = 100
5	22	Lingual Marginal ridge mesial—33 43	8 = 38,1	4 = 50,0
6	29	Lingual Marginal ridge distal—33 43	8 = 38,1	6 = 75,0
7	92	Number of main cusps: 3 cusps—35 45	8 = 38,1	4 = 50,0
8	109	Fissure pattern: half-round—34 44	11 = 52,4	2 = 18,2
9	112	Fissure pattern: double pit—34 44	11 = 52,4	9 = 81,8
10	115	Fissure pattern: half-round—35 45	8 = 38,1	2 = 25,0
11	117	Fissure pattern: y-shaped—35 45	8 = 38,1	4 = 50,0
12	118	Fissure pattern: double pit—35 45	8 = 38,1	2 = 25,0
13	135	Grooved marginal ridges: mesial—34 44	11 = 52,4	5 = 45,5
14	212	Number of main cusps: 5 cusps—36 46	15 = 71,4	14 = 93,3
15	215	Number of main cusps: 4 cusps—37 47	16 = 76,2	15 = 93,8
16	219	Number of main cusps: 4 cusps—38 48	11 = 52,4	8 = 72,7
17	220	Number of main cusps: 5 cusps—38 48	11 = 52,4	4 = 36,4
18	249	Fissure pattern: y—36 46	6 = 28,6	4 = 66,7
19	251	Fissure pattern: x—36 46	6 = 28,6	2 = 33,3
20	253	Fissure pattern: y—37 47	12 = 57,1	3 = 25,0
21	254	Fissure pattern: +- 37 47	12 = 57,1	2 = 16,7
22	255	Fissure pattern: x—37 47	12 = 57,1	9 = 75,0
23	257	Fissure pattern: y—38 48	6 = 28,6	2 = 33,3
24	259	Fissure pattern: x—38 48	6 = 28,6	5 = 83,3
25	526	Entoconulid—38 48	12 = 57,1	2 = 16,7
26	527	Foramina molaria—36 46	12 = 57,1	6 = 50,0
27	528	Foramina molaria—37 47	14 = 66,7	8 = 57,1
28	529	Foramina molaria—38 48	12 = 57,1	4 = 33,3
29	555	Paramolar tubercle: microform—38 48	13 = 61,9	4 = 30,8

^1^ for a detailed description of the traits see [64].

## Results

### Search for increased frequencies (Strategy 1)

In our sample population, four of the 29 traits of Table 2 show a distinctly increased relative frequency compared to our reference population. These are shown in Table 3. This result suggests that our sample population may be somewhat different from the reference population and that there are first hints to biological relationships within the sample population.

**Table 3:** Four traits with increased frequency in Kfar HaHoresh and their relative frequencies within the population of Kfar HaHoresh and within the reference population.

Traits	Frequency within Kfar HaHoresh (n = 21)			Frequency outside Kfar HaHoresh (n = 251)		
	evaluable	present	relative frequency in %	Evaluable	present	relative frequency in %
t 92	8	4	50,0	102	26	25,5
t 220	11	4	36,4	93	10	10,8
t 526	12	2	16,7	80	6	7,5
t 555	13	4	30,8	89	18	20,2

### Search for pairwise similarities (Strategy 2)

The consideration of pairwise similarities requires the preselection of suitable traits. If all traits were used, an existing biological similarity based on a few traits would be masked by the noise of all other traits. As the increased frequency of a trait in comparison to a reference population may be a hint to biological relationships among the carriers of the trait, we base our analysis of pairwise similarity on the four traits identified by strategy 1.

For each pair of individuals, we first consider the number of traits discernible in both individuals and present in at least one of them. Then we determine the fraction of traits actually present in both, known as Jaccard index. Table 4 shows all pairs of individuals with at least two of the four traits present in both, ordered according to the Jaccard index.

**Table 4:** Results of the search for pairwise similarities.

Individual 1- Individual 2	Number of traits discernible in both individuals and present in at least one-Number of traits present in both individuals	Jaccard index in %
ind. 4 – ind. 7	3–3	100
ind. 2 – ind. 28	2–2	100
ind. 2 – ind. 24	2–1	50
ind. 24 – ind. 5	2–1	50
ind. 16 – ind. 24	2–1	50
ind. 16 – ind. 7	3–1	33
ind. 16 – ind. 4	3–1	33
ind. 2 – ind. 4	3–1	33
ind. 2 – ind. 7	3–1	33
ind. 24 – ind. 28	3–1	33
ind. 28 – ind. 7	4–1	25
ind. 28 – ind. 4	4–1	25
ind. 24 – ind. 4	4–1	25
ind. 24 – ind. 7	4–1	25

We could identify two pairs of individuals with a Jaccard index of 100% (Table 4). According to the sex and age analysis of these individuals by Tal L. Simmons, both pairs comprise juvenile to adult women. In the following, we focus only on these two pairs. Because of the small number of traits used, it remains unclear whether Jaccard indices of 50% or less can be interpreted as evidence of biological similarities.

Among the four traits of Table 3, individuals 4 and 7 have the traits t 92, t 220, and t 526 in common, and individuals 2 and 28 have the traits t 220 and t 555 in common. The heritability of the analyzed traits considerably determines the relevance of the similarities of traits as evidence of a genetic relationship of the analyzed pairs. The number of cusps of the second premolar of the mandible (t 92) and of the third molar in the mandible (t 220) is strongly variable. As with many norm variations of teeth, ethnic differences exist and thus accumulations in families may develop [58].

The relevance of additional cusps of the lower molars (traits: t 526 and t 555) is slightly stronger than for the normal variation of cusps. The population specific frequencies and the genetic basis of the former two traits have been studied in a better way. For the Tuberculum paramolare (syn. Protostylid) (t 526) the genetic information is well documented. This trait is highly variable between minus and plus variations on the mesio-buccal cusp of all three molars [64]. An increased frequency of the Tub. paramolare has been observed consistently by several authors within Mongoloid populations [75–76]. Considerable ethnic differences in the frequency of this trait [77–78], an accumulation within families [79–80] and the results of twin-analyses [81] underline the importance of genetic inheritance.

The entoconulid (t 555; syn. Tuberculum sextum, cusp-6), an accessory cusp, which is found on the distal marginal ridge between the hypoconulid and the entoconid of lower molars, shows similar characteristics in population genetics [64]. Like the protostylid, cusp-6 is rarely found in Caucasian and Black African populations, whereas it is significantly higher within mongoloid Asian populations and in Australian Aborigines [77, 82]. According to twin [81, 83] and family studies [79] there is no doubt about the genetic basis of this trait.

### Search for conspicuous groups of individuals within the data matrix (Strategy 3)

Our strategy of a systematic search for blocks in the data matrix, which are characterized by the shared presence of rare traits in a subgroup of individuals, is described in detail elsewhere [64]. In the analysis presented here, all 29 traits shown in Table 2 were included. We analyzed the individuals of Kfar HaHoresh and those belonging to the reference population together, such that “rare” refers to a low frequency in the joint data set. However, we restricted our search for subgroups to individuals from Kfar HaHoresh. This approach allows us to identify two group structures (A and B).

#### Group A

Group A consists of six individuals with strong similarities. These are three individuals of the age range between 15 and 25 years (ind. 2, 4, and 7). All three were probably female. Additionally, a 25-30-year-old woman (ind. 40), a male adolescent (ind. 24) and an older child (ind. 13; infans II) belong to this cluster. Their biological relationship is demonstrated by similar anatomical norm variations and anomalies of the teeth crowns in the mandible. In accordance to the results of the similarity analysis, the strongest resemblance exists between the juvenile ind. 4 and the adult ind. 7 sharing five of the six group defining traits. Both individuals were probably female. These two key-individuals have the following traits in common: t 92 (number of main cusp [3] on lower second premolars), t 135 (grooved marginal ridges on lower first premolars, mesial), t 220 (number of main cusps [5] on third molars in the lower jaw), t 526 (entoconulid of third molars in the lower jaw) and t 528 (foramen molare of the second molars in the lower jaw). It is by the last trait t 528 and by one additional trait each, that the other individuals of Group A are identified as similar to the ind. 4 and ind. 7: Ind. 40 shows the trait t 528 and the trait t 254 (cross-pattern of the fissures of the chewing surfaces of the second molars in the mandible) and is thus similar only to ind. 7. Ind. 24, the male adolescent, is related to both individuals by the traits t 528 and t 92. Ind. 2 has the traits t 528 and t 220 in common with both key-individuals and traits t 528 and t 135 relates the only child of the group (ind. 13) to both individuals 4 and 7 (Table 5).

**Table 5:** Distribution of common traits in Group A.

Ind.	t 92	t 135	t 220	t 254	t 526	t 528	Age group	Sex
2	??	??	?+	?-	?-	?+	Adult	Female?
4	?+	-+	-+	—	++	++	Juvenile	Female?
7	-+	?+	++	+-	+-	++	Adult	Female?
13	??	?+	??	-?	??	+?	Infans II	Indet
24	++	—	-?	-?	-?	++	Juvenile	Male
40	-?	-?	-?	+?	-?	+?	Adult	Female

+:present

-:absent

?:indiscernible

illustrated for the teeth of the left and the right side of each lower jaw.

The differences of frequency between populations evidence the heritability of traits t 220 und t 254 [58]. For trait t 528 population genetics as well as familial specificities also speak in favour of the heritability of the trait [84]. We have already demonstrated in detail the heritability of trait t 526 (s. p. 15).

Table 6 displays the relative frequencies of traits in Group A compared to the other individuals of Kfar HaHoresh and to the joint data set. It shows that two of the six traits characteristic for Group A can be found in Kfar HaHoresh exclusively in Group A. Two other defining traits of Group A can be found only in one further individual outside Group A. The relative frequencies of the group defining features outside Group A in Kfar HaHoresh or outside of Kfar HaHoresh are in the very low to middle ranges, whereby relative frequencies of up to 10% are considered very low, between 10% and 30% as low and frequencies between 30% and 60% are considered in the middle ranges. Except for the two traits with a 0 frequency, the relative frequencies outside of group A in Kfar HaHoresh and outside Kfar HaHoresh are rather similar.

**Table 6:** Frequencies of group defining traits for Group A.

Traits	Kfar HaHoresh (n = 21)								reference populations (n = 251)			
	*a) n inside group A*				*b) n outside group A*				*c) n outside KHH*			
	+	-	?	*Rel*. *Fre %*	+	-	?	*Rel*. *Fre %*	+	-	?	*Rel*. *Fre %*
t 92	3	1	2	75.0	1	3	11	25.0	26	76	149	25.5
t 135	3	2	1	66.7	2	4	9	33.7	32	57	162	36.0
t 220	3	2	1	66.7	1	5	9	16.7	10	83	158	10.8
t 254	2	4	0	33.7	0	6	9	0	14	81	156	14.7
t 526	2	3	1	40.0	0	7	8	0	6	74	171	7.5
t 528	6	0	0	100	2	6	7	25.0	39	79	133	33.1

KHH = Kfar HaHoresh, n = absolute number, rel. fre. = relative frequency of presence.

+:present at the right or left side of the lower jaw

-:absent at all discernible sides

?:indiscernible at the right and left side of the lower jaw

#### Group B

In Group B seven individuals share partially five defining traits (ind. 2, 4, 7, 13, 24, 28 and 40). Two individuals show 2 of the 5 traits, four individuals 3 of the 5 traits, and one individual (ind. 24) 4 of the 5 traits. A close relationship between ind. 2 and 28 has already been demonstrated by the similarity analysis. Most of the individuals of Group B overlap with those of Group A, only one new individual is included: Ind. 28, an adult, whose sex has been determined by Simmons as”probably female“. This individual is actually indiscernible in the trait t 528, the most decisive trait of group A (Table 7). Most of the defining traits have already been described for Group A. New common traits are t 22 (lingual marginal ridge mesial on the canines in the lower jaw) and t 555 (paramolar tubercle microform on the third molars of the lower jaw). The heritability of both traits has already been described (s. p. 13–14). Actually, these two new traits together with t 220 link ind. 28 to the rest of the group: t 22 links ind. 18. to ind. 13, 24, and 40; she shares trait t 220 with ind. 2, 4, and 7 and trait t 555 with ind. 2 and 24, thus sharing two traits with ind. 2 and 24.

**Table 7:** Distribution of common traits in Group B.

Ind.	t22	t92	t220	t528	t555	Age group	Sex
2	??	??	?+	?+	?+	Adult	Female?
4	??	?+	-+	++	-?	Juvenile	Female?
7	—	-+	++	++	—	Adult	Female?
13	++	??	??	+?	??	Infans II	?
24	+?	++	-?	++	+?	Juvenile	Male
28	++	—	+-	??	+-	Adult	Female?
40	+?	-?	-?	+?	-?	Adult	Female

+:present

-:absent

?:indiscernible

demonstrated for the teeth of the left and the right side of each lower jaw.

As for Group A, the relative frequencies of traits outside Group B in Kfar HaHoresh are closer to the frequencies in the joint data set than to those inside Group B (Table 8). The new trait t 22 is absent outside Group B in Kfar HaHoresh and t 555 is expressed only once outside Group B in Kfar HaHoresh. The relative frequencies of the group defining traits outside Group B in Kfar HaHoresh and outside of Kfar HaHoresh are in the very low and low ranges, and only in two cases do they slightly cross the border to the middle ranges (33.3% and 33.1%) respectively.

**Table 8:** Frequencies of group defining traits for Group B.

Traits	Kfar HaHoresh (n = 21)								reference populations (n = 251)			
	*a) n inside group B*				*b) n outside group B*				*c) n outside KHH*			
	+	-	?	*Rel*. *Fre %*	+	-	?	*Rel*. *Fre %*	+	-	?	*Rel*. *Fre %*
t22	4	1	2	80.0	0	3	11	0	23	62	166	27.1
t92	3	2	2	60.0	1	2	11	33.3	26	76	149	25.5
t220	4	2	1	66.7	0	5	9	0	10	83	158	10.8
t528	6	0	1	100	2	6	6	25.0	39	79	133	33.1
t555	3	3	1	50.0	1	6	7	16.7	18	71	162	20.2

KHH = Kfar HaHoresh, n = absolute number, rel. fre. = relative frequency of presence.

+: present at the right or left side of the lower jaw

-: absent at all discernible sides

?: indiscernible at the right and left side of the lower jaw

## Discussion

Both the analysis for the pairwise similarities as well as the systematic search for group structures indicate that the strongest similarity exists between ind. 4 and 7. Their biological relationship is represented by two common traits in the similarity analysis and by five common traits within Group A, the strongest relationship within this group. The same three common traits for these individuals are replicated in Group B (Tables 7 and 5). Interestingly, both individuals and ind. 2 and 13 which also belong to Group A and B, were buried in Loc. 1003. However, it should be stressed that more than half of the investigated individuals came from that grave (Fig 2).

The relationship in Group A is based on six common traits, of which five are shared by ind. 4 and 7 and two with each of the other individuals of that group (ind. 2, 13, 24 and 40). These traits comprise four norm variations and two anomalies of the teeth crown of the premolars and molars in the mandible. Interestingly, Group A is dominated by four women. Simmons et al. [44] determined three as probably female and one as certainly female. The age ranges of the women are between 15 and 30 years. The only male individual of that group is an adolescent. Neither one of the six male individuals buried in Loc. 1003 nor any other male adult (n = 4) belong to this group (Table 1 and Figs 2 and 3). One of the three investigated children (ind. 13), also buried in Loc. 1003, shares two traits with ind. 4 and 7, and one trait with ind. 2. For the other children only 2 teeth of the mandible could be analyzed, so the biological relationship of these individuals is difficult to assess.

In Group B, another probable female adult (ind. 28) is clustered together with the other six individuals known already from Group A. Another adult female (ind. 39) does not show significant similarities with the individuals of both groups.

The individuals of groups A and B are clearly separated from the other individuals buried at the site with respect to the frequency of common traits (see Tables 6 and 8). For the individuals of Kfar HaHoresh outside of groups A and B, the frequency of traits typical for groups A and B has more similarities with the joint data set, than with the frequency within the groups A and/or B.

When interpreting the results, the following must be taken into account: a large number of evaluable teeth in an individual increases its likelihood of probable biological relations to other individuals. For example, four out of the six individuals with at least 10 evaluable teeth show up in at least one of the two groups. However, the fact that two of them did not show up, actually illustrates that it is still possible to differentiate between individuals. For all 21 investigated individuals, with the exception of three cases, only the teeth of the mandible could be analyzed. It is thus not surprising that for the assignment to Group A and B only traits of the mandible were relevant.

Interestingly, the spatial distribution of the investigated individuals shows that four individuals of the groups with significant similarities came from Loc. 1003. The other individuals came either from Loc. 1155 (ind. 24 and 28) contemporary with Loc. 1003 or from Loc. 1157 (ind. 40), which is more ancient than Loc. 1003. The time elapsed between these phases determines whether these similarities can be interpreted as close biological relationship or whether the common traits were characteristic for a genetically stable population. Burials of other areas with investigated individuals did not show significant similarities (Loc. 1110, 1152, 1211, 1156). Though roughly contemporary, the woman (ind. 39) who does not share common traits on teeth with the other individuals is spatially separated from the other related individuals (ind. 24 and 28) of Loc. 1155/1352/1353/1373 by the plastered surface Loc. 1027.

A final assessment of our results for the reconstruction of the social structure is difficult. First of all, only 21 of the about 80 excavated individuals could be investigated in our study on biological relationships. The preservation and completeness of the investigated jaws and teeth was less than suboptimal. Concerning the diagnosis of age and sex, some determinations are uncertain and some are not concurrent in the analyses of the different investigators (cf. [46]; Simmons pers. obs.).

We can thus suggest tentatively that according to the sex and age determinations of Simmons, young adolescent to adult females show closer biological relationships with each other and with the–admittedly small sample investigated–sub-adults, than with the male individuals buried on-site. In contrast, none of the adult males of any phase or any location show significant similarities in the evaluable traits. This is all the more striking because their number outweighs those of the females (10:6; Fig 2) and because two of the plastered skulls of Kfar HaHoresh were determined as male and probably male respectively [43,46]. Thus, the male individuals do not only display higher genetic heterogeneity, but some male individuals also underwent special treatment after death compared to the female individuals. The latter has also been suggested for other sites in the southern Levant during the Middle and Late PPNB [7, 85].

These results differ from the observations made at the contemporary, Late PPNB village of Basta in southern Jordan, where very close familial relationships were suggested akin to endogamy [31] and at chronologically later Çatalhöyük in central Anatolia, where the female skeletons show a higher degree of variability compared to the male individuals, which was interpreted as evidence for immigration of female individuals [30]. There could be different reasons for the higher variability of the male individuals from Kfar HaHoresh. However, due to the incomplete preservation of the skeletons, the age and sex determinations remain uncertain and the representativeness of our results should be considered with caution. It would be premature to interpret our results as evidence for matrilocality without any data on the mobility and the possible origins of the individuals.

## Conclusion

Our results point to the existence of biological relationships among some of the analyzed individuals, above all among females and sub-adults. They also suggest that biological relationships may coincide partially with spatial closeness at Kfar HaHoresh. Taken together, these conclusions suggest that matrilocal biological relationships may have played a role in the burial practice. The interpretation of the presence or absence of family oriented burial practices depends mainly on the chronology of the burials. For the four related ind. 2, 4, 7, and 13 (3 females and a child), all buried in Loc. 1003, within a short time span, family relations might be the key explanation. However, it should be stressed that the six male individuals buried in the same location did not show biological relationships in the investigated data.

If the burials of the individuals were spread over a larger time span, as the other individual related to groups A and B: ind. 40, we can only conclude that a subgroup of individuals, which was genetically stable over time, partially used the same burial site. Whether this subgroup of the population actually existed, or whether the data merely represents the local population for a certain time period during the use of the site, which was then exchanged by a genetically different population, remains unknown.

If the suggested sex determinations hold true, matrilocal burial patterns may be postulated at Kfar HaHoresh, at least during the late period of its use. A final conclusion on group homogeneities and residence patterns can only be made once the final analyses of the whole skeletal corpus from Kfar HaHoresh are available.

## Supporting Information

S1 Fig Photos of the teeth and mandibles of individuals 1 to 7 (photos: J. Kranzbühler). (TIF)

S2 Fig Photos of the teeth and mandibles of individuals 9–24 (photos: J. Kranzbühler). (TIF)

S3 Fig Photos of the teeth and mandibles of individuals 25–40 (photos: J. Kranzbühler). (TIF)

S1 Table Sex and age determinations of individuals studied by Tal L. Simmons. M = mandible; x = (parts of) both left and right mandible; yrs = years.
(DOCX)
