# Supplementary figures and images for: Insights into the Social Structure of the PPNB Site of Kfar HaHoresh, Israel, Based on Dental Remains
Source: PLoS One. 2015 Sep 16;10(9):e0134528. doi: 10.1371/journal.pone.0134528 (PMC4573520; doi:10.1371/journal.pone.0134528)

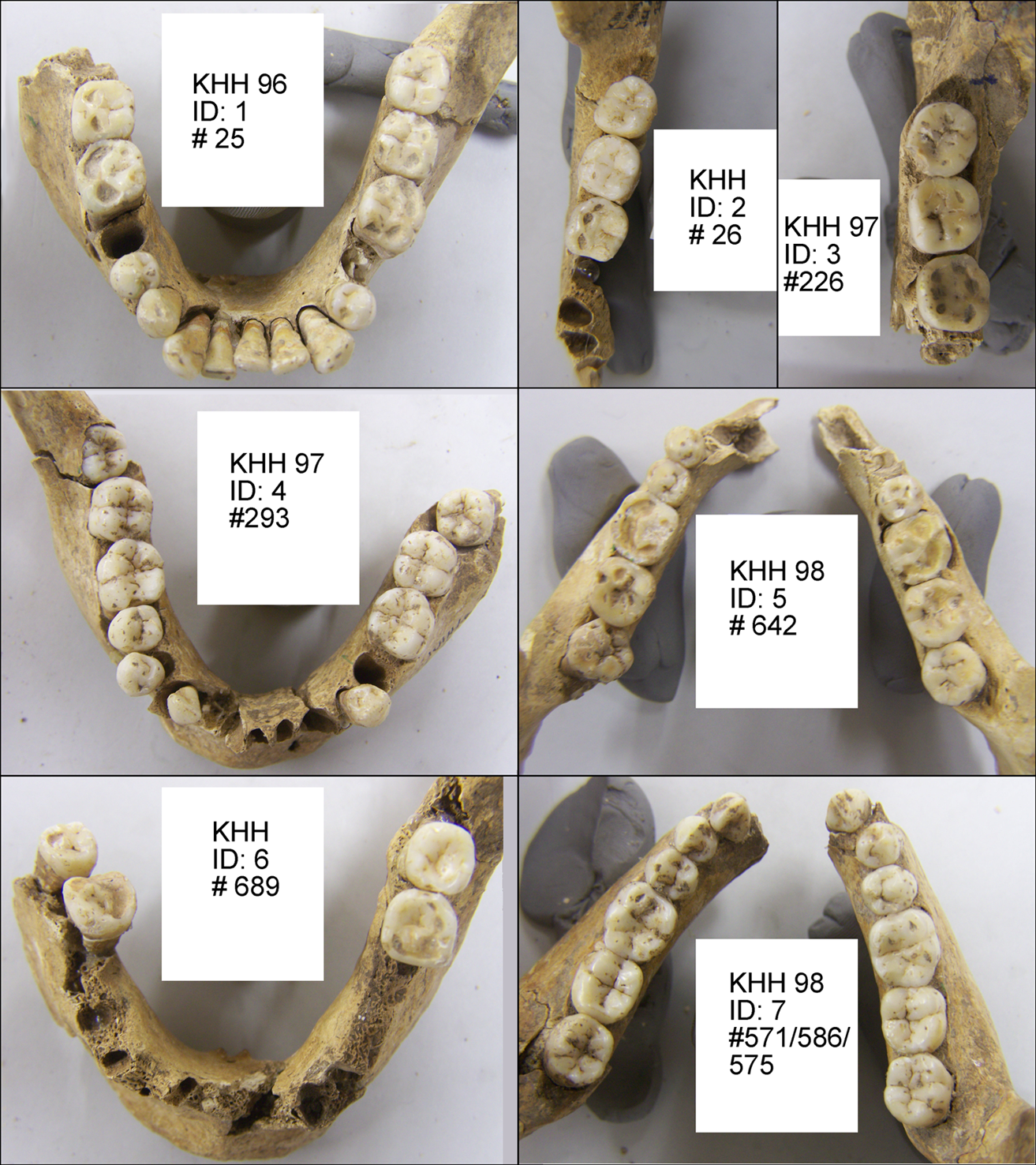

Supplement: S1 Fig — (TIF) [file pone.0134528.s001.tif]

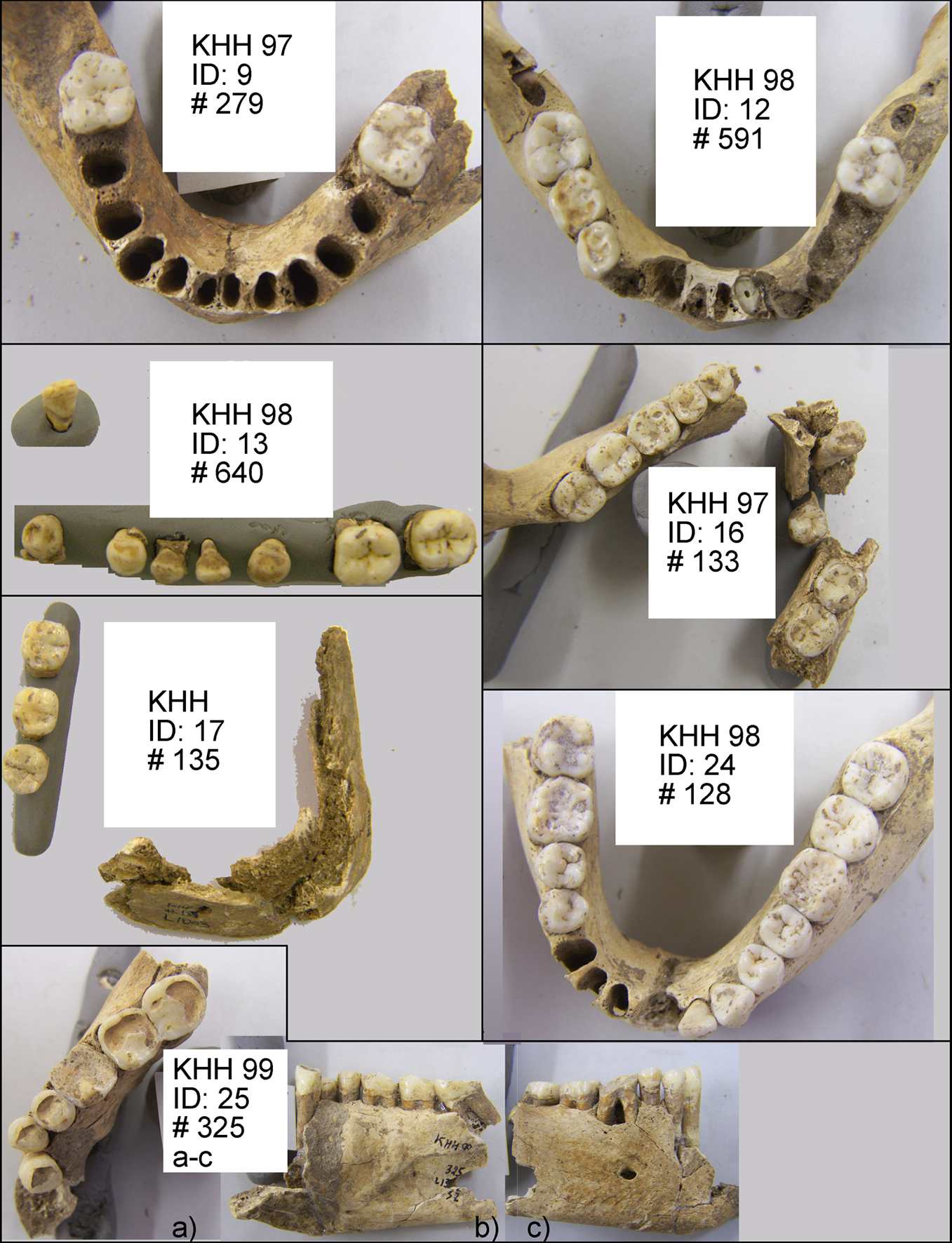

Supplement: S2 Fig — (TIF) [file pone.0134528.s002.tif]

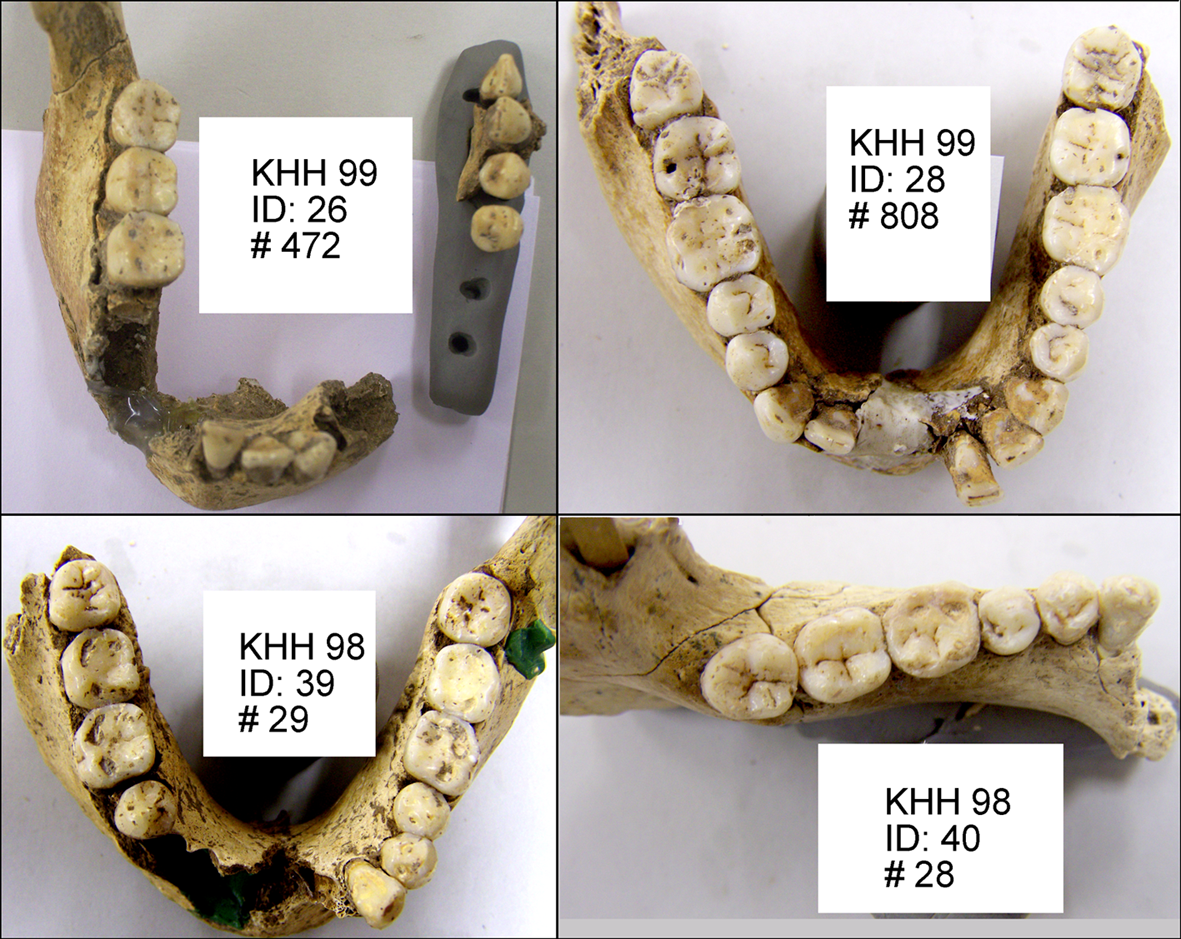

Supplement: S3 Fig — (TIF) [file pone.0134528.s003.tif]
