# Supplementary material for: Insights into the Social Structure of the PPNB Site of Kfar HaHoresh, Israel, Based on Dental Remains
Source: PLoS One. 2015 Sep 16;10(9):e0134528. doi: 10.1371/journal.pone.0134528 (PMC4573520; doi:10.1371/journal.pone.0134528)
Supplement: S1 Table — M = mandible; x = (parts of) both left and right mandible; yrs = years. (DOCX) [file pone.0134528.s004.docx]

| **ID N°** | **Specimen** | **Locus** | **Square** | **Bone** | **Side** | **Part** | **Sex** | **Criteria (sex)** | **Age (yrs)** | **Criteria (age)** |
| --- | --- | --- | --- | --- | --- | --- | --- | --- | --- | --- |
| 1 | #25 | 1003 | J51d | M | X | left corpus and ramus, right corpus; right condyle | male? | double pointed mental eminence | 25-30 | dental wear |
| 2 | #26 | 1003 | J51d | M | X | fragment of right corpus and ramus; with fragments of left ramus including coronoid process and lingula | female? | gracile | 20-25 | dental wear |
| 3 | #226 | 1003 | K52 | M | X | fragment (molar area only) of left corpus and lower ramus | male? | medial pterygoid insertions robust | 25-30 | dental wear |
| 4 | #293 | 1003 | J51c | M | X | right ramus and corpus, left corpus; left condyle | female? | pointed mental eminence | 15-20 | M2's in occlusion and slightly worn; M3's unerupted |
| 5 | #642 | 1003 | K52 | M | X | right and left rami and corpi without symphysis area | male? | medial pterygoid insertions robust | 25-30 | dental wear |
| 6 | #689 | 1003 |  | M | X | left corpus and ramus, right corpus | male? | double pointed mental eminence; masseter attachment | 25-30 | dental wear |
| 7 | #571/575 | 1003 | J51a | M | X | right and left rami and corpi without symphyseal area | female? | gracile | 15-25 | M3's erupted and little wear |
| 9 | #279 | 1003 | J52a | M | X | right corpus and ramus (without condyle) and left corpus | ? | - | Adult | dental wear |
| 12 | #591 | 1003 | J52a | M | X | right and left rami and corpi without symphyseal area | ? | - | subadult (ca. 7-9) | dental eruption (M1 in occlusion: M2 in crypt with some root development) |
| 13 | #640 | 1003 | J52 | M | X | fragments of left ramus, including condyle and coronoid process; mental eminence | ? | - | subadult (4-6) | nearly complete root of M1's and incisors |
| 22 | #4 | 1110 | F61d | M | right | corpus | ? | - | 35-40 | Dental attrition |
| 25 | #325 | 1352 |  | M | right | corpus fragment | ? | - | Adult (35-45) | M3 erupted; extreme tooth wear |
| 26 | #472 | 1353 | Q58a/b | M | Partial | partial right ramus, right corpus, and left partial corpus | ? | - | adult  (ca. 25-30) | dental wear |
| 28 | #808 | 1373 |  | M | X | complete corpus | female? | pointed mental eminence | adult  (25-35) | M3's erupted (tooth wear) |
| 40 | #28 | 1157 | S56/R56 | M | right | fragment with M2 | ? | - | 25-30 | dental attrition |
| 40 | #28 | 1157 | S56/R56 | M | left | complete half with Canine-M3 | ? | - | 25-30 | dental attrition |
